# 肺部磨玻璃影的诊断与治疗进展

**DOI:** 10.3779/j.issn.1009-3419.2016.11.09

**Published:** 2016-11-20

**Authors:** 悦 赵, 瑞 王, 海泉 陈

**Affiliations:** 1 200032 上海，复旦大学附属肿瘤医院胸外科 Department of Thoracic Surgery, Fudan University Shanghai Cancer Center, Shanghai 200032, China; 2 200032 上海，复旦大学上海医学院肿瘤学系 Department of Oncology, Shanghai Medical College, Fudan University, Shanghai 200032, China

**Keywords:** 肺部磨玻璃影, 诊断, 治疗, 进展, Ground-glass opacity, Diagnosis, Treatment, Progression

## Abstract

近年来，肺部磨玻璃影（ground-glass opacity, GGO）逐渐得到了肿瘤科和胸外科医生的普遍关注。GGO是指肺部CT表现为密度轻微增加，增加程度小于实性改变，呈模糊的云雾状，并可见其内血管和支气管纹理。GGO多数情况下呈惰性，但也可进一步发展为肺腺癌，这使其治疗方案的选择颇为棘手；近年来GGO发现率的日益增加也使其关注度得到大大提升。许多报道都从组织学、放射诊断学、治疗学等多个方面对GGO的诊治进行了探讨。本文综述了近10年来学界对GGO的诊断和处理的进展，希望临床医生能更好地认识这个问题，在临床工作中收集并总结更多循证学证据，以指导未来的临床诊治方案的选择。

## Abstract (en)

Pulmonary ground-glass opacity (GGO) has raised increasing attention of clinical oncologists and thoracic surgeons in recent years. GGO appears as hazy increased opacity of lung, with preservation of bronchial and vascular margins. GGO is less opaque than consolidation, in which bronchovascular margins are obscured. In most cases, GGOs are indolent, while in other cases, they may grow malignant. This pattern makes it difficult to find a standard way to treat patients with GGO. Moreover, with the improvement of technology, more and more GGOs are found in patients' computed tomgraphy (CT) scan, making it a global problem. In the literature, many clinicians have reported their findings on this topic from histologic, radiologic and therapeutic perspectives. In this study, we reviewed the development on this topic in recent 10 years. We hope our study can be helpful for clinicians to better understand this problem, collect more data in their clinical work and most importantly, let evidence guide our future therapeutic choices.

## 1 前言

近年来，由于高分辨率计算机断层扫描（computed tomography, CT）的广泛应用，医生可以查出更多的早起肺癌，也使“肺部磨玻璃影”一词得到了广泛关注。肺部磨玻璃影（ground-glass opacity, GGO）被定义为“模糊增高的肺部阴影，可见其内部的支气管和血管纹理”^[1]^，与实变不同，实变指的是“肺间质密度的均一增加，并使其内的血管和气道轮廓变得模糊不清”^[1]^。根据肺部磨玻璃影内部成分均一程度的不同，GGO可分为单纯磨玻璃影（pure ground-glass opacity, pGGO）和混合性磨玻璃影（mixed ground-glass opacity, mGGO）两种类型。此前有报道^[2]^称pGGO的恶性率约18%，而mGGO的恶性率约为64%。而以GGO为表现的早期肺癌的术后5年生存率高达100%^[3]^。因为其有着较高的恶变率和早期诊疗的高生存率，以及其在影像学上难以发现良恶性程度的特点，使得GGO的诊治成为了当今学术界的热门话题。

## 2 GGO的流行病学特点

多种肺部疾病可以表现为肺部磨玻璃影，且根据肺部病变的大小、受累范围的不同，有不同的诊断提示。GGO结节是肺部局限性磨玻璃影表现的一种。根据CT图像显示，有相当一部分肺部结节都表现为GGO结节，并且查出GGO结节的患者的临床特点往往不同于确诊肺癌的患者^[4]^。往往在亚洲人群、女性、以及不吸烟的人群中更易查出GGO结节，这个现象已被报道在日本与韩国的不少研究中^[4]^，这一流行病学特点与肺腺癌[尤其是有表皮生长因子受体（epidermal growth factor receptor, *EGFR*）突变的肺腺癌]的流行病学特点颇为相似^[5, 6]^，或许二者之间存在着某种联系。

## 3 GGO的影像学特点

GGO在CT图像上显示为“模糊增高的肺部阴影，可见其内部的支气管和血管纹理”^[1]^。在肺部CT中，患者的病灶中可以同时出现磨玻璃影和实变影，因此，Suzuki等^[7]^于2006年根据磨玻璃影和实变在同一病灶中所占比例的不同，将肺腺癌患者CT图像上表现的磨玻璃样病变进一步分成了六种类型，分别为：1型：单纯性GGO；2型：半实变型（间质均一性的密度增高）；3型：光晕型（病灶中央是实性的部分，周围是由GGO构成的光晕）；4型：混合性GGO（病灶含有GGO和实性部分，并伴有支气管充气征）；5型：伴有GGO的实性型（GGO所占比例 < 50%）；6型：完全实性型。其中，Ⅰ期患者在六种类型中所占比例分别为100%、100%、96.0%、100%、94.4%以及72.6%；而Ⅲa期及以上患者在六种类型中所占比例分别为0、0、0、0、5.6%以及11.7%。这种更加精细的分型为GGO的发展程度的诊断以及对其分期的判断提供了可能。根据GGO结节的恶性程度不同而有不同的Lung-RADS分级，其中，没有结节或有钙化结节为1级； < 6 mm的实性/部分实性结节、 < 20 mm的纯GGO结节为2级；≥6 mm但 < 8 mm的实性结节、≥6 mm但实性成分 < 6 mm的部分实性结节、以及≥20 mm的纯GGO结节为3级；≥8 mm但 < 15 mm的实性结节、≥8 mm且实性成分≥6 mm但 < 8 mm的部分实性结节为4A级；≥15 mm的实性结节、实性成分≥8 mm的部分实性结节为4B级；有着其他特殊征象、影像学怀疑是癌症的3、4级结节为4X级^[8]^。随着等级的提高，结节为恶性的机会逐渐增大。表现为GGO的病灶也可以在随访过程中发生变化。一个韩国的报道^[9]^称，37%的单纯性GGO和48%的混合性GGO在3个月的时间内缩小或消失了。然而，也有一部分研究表明GGO是可以随着时间的推移而增长的，几个来自日本的研究证实了这一点：Hiramatsu等^[10]^于2008年发表文章称26%的GGO增长了至少2 mm；Matsuguma等^[11]^和Kobayashi等^[12]^的报道分别显示了有41%和29%的混合性GGO在随访过程中观察到明显增长；无独有偶，来自韩国的几项研究也用翔实的数据支持了这一说法：Chang等^[13]^发表文章称，12%的单纯性GGO在随访过程中发生了明显的增长；Lee等^[14]^在2013年发表的一篇论文中运用统计分析方法指出，混合性GGO、初诊时较大的病灶以及高龄患者是GGO随访过程中增长的独立高危因素。

## 4 GGO的临床特点

放射学诊断与病理学诊断的结果未必相同，而且并不是一对一的对应关系。在放射学诊断（胸部CT或增强CT）上表现为GGO的病灶，其本质可有多种可能，可以是恶性的病变，如肺部原位癌（adenocarcinoma *in situ*, AIS）、微侵袭性肺腺癌（minimally invasive adenocarcinoma, MIA）、侵袭性肺腺癌（invasive adenocarcinoma, IA）等，也可以是非恶性的病变，如肺部非典型腺癌样增生（atypical adenomatous hyperplasia, AAH）、炎症、纤维化等以及其他的良性病变^[13, 15-22]^。

早在2009年，Godoy等就指出了不同病理学类型与GGO结节影像学表现之间的关系： < 5 mm的纯GGO几乎全部为不典型腺瘤样增生，而细支气管肺泡癌（细支气管肺泡癌这一名词当时仍在使用）可以表现为纯GGO或可能伴有实性成分的GGO；而 > 1 cm的GGO应当被认为是细支气管肺泡癌或侵袭性腺癌，需要进行随访和干预^[23]^。

El-Sherief等^[24]^在2014年根据不同临床和病理学特点的GGO在影像学表现上的不同，将GGO分成了7大类，分别为：①弥漫性GGO；②小叶中心型GGO；③结节状GGO；④镶嵌状GGO；⑤碎石路样GGO；⑥光晕征；⑦反光晕征。不同类型的GGO所对应的临床和病理学特点见表 1。

**1:** 肺部磨玻璃影的分类及对应的临床病理学特点 Classification and clinicopathological characteristics of GGO

Type of GGO	Clinicopathological characteristics
Diffuse GGO	(1) Acute dyspnea (including cardiogenic pulmonary edema, diffuse alveolar hemorrhage and acute interstitial pneumonia); (2) Slowly progressive dyspnea (including nonspecific interstitial pneumonia, desquamative interstitial pneumonia, lymphocytic interstitial pneumonia and radiation pneumonitis); (3) Diffuse GGO in immunocompromised patients (pneumocystis pneumonia and viral pneumonias).
Crazy paving	(1) Pulmonary alveolar proteinosis.
Mosaic attenuation	(1) Patchy interstitial disease;
	(2) Obliterative small-airways disease;
	(3) Occlusive vascular disease.
Centrilobular GGO	(1) Aspiration; (2) Respiratory bronchiolitis; (3) Subacute hypersensitivity pneumonitis; (4) Metastatic pulmonary calcification; (5) Pulmonary cholesterol granulomas.
Persistent nodular GGO	(1) AAH; (2) AIS and MIA.
Halo sign	(1) Hemorrhagic nodules or masses; (2) pulmonary infiltration by tumor; (3) inflammatory disorders.
Reversed halo sign	(1) Cryptogenic organizing pneumonia.
GGO: ground-glass opacity; AAH: atypical adenomatous hyperplasia; AIS: adenocarcinoma in situ; MIA: minimal invasive adenocarcinoma.	

炎症等良性病灶可随时间的推移逐渐缩小甚至消失，而恶性病灶随着时间的推移将逐渐增大，甚至发生淋巴结转移和远处转移^[25]^。可是其在CT图像上都表现为GGO（或GGO结节），因此，如何早期诊断GGO、确定其性质，并根据其性质进行相应的治疗，成为了目前临床上亟待解决的问题。

## 5 GGO的诊断和治疗方案的选择

既然表现为GGO的病灶的性质有各种可能，如何确定表现为GGO的病灶的性质、并针对其性质进行相应的治疗，便显得格外重要。而由于表现为GGO的病灶往往较小（< 10 mm），很难用细针抽吸的方式取得活检标本，这使得确定其良恶性变得更为困难。基于临床医生的观察，多数GGO在很长一段时间内都会保持不变，所以许多临床医生都忽视了GGO的重要性，正如同他们对甲状腺微小结节所持的态度那样^[4]^。而也有一部分医生开始对GGO产生警惕，认为不能忽视其长期的增长性。目前学界普遍认为，对CT图像上的病灶表现为GGO的患者应当进行至少为期3年的随访，一旦病灶有增长的现象或是实变程度增加，则考虑外科手术干预^[26]^。Shinohara等^[27]^于2015年发表的一篇病例报告显然支持了这一观点，他们把1例CT表现为GGO结节的患者当作肺结核来处理，起初一切顺利，直到6年后患者出现呼吸系统症状，复查发现结节变大，手术时发现广泛的胸膜播散，成为T1N0M1的Ⅳ期肺癌患者，失去了治疗的绝佳机会。这不得不使我们感到惋惜，并不得不重新认识CT图像上表现为GGO结节的病灶的重要性。那么，究竟什么样的患者适合手术？一个被广泛采纳的由Fleischner协会指出的手术指征为：①直径 > 15 mm的单纯性GGO；②实性成分 > 5 mm的混合性GGO^[28]^。也有学者^[29]^指出，在随访过程中发现的明显增大（> 2 mm）的GGO或是病灶中含有实变的成分即可考虑手术。在手术术式的选择方面，学界更倾向于对直径在10 mm-20 mm之间的病灶进行局限性的切除（如亚肺叶切除、肺段切除和楔形切除），局限性切除相比标准的肺叶切除术得到的结果相似^[30]^，但却可以更多地保留患者的肺功能，因而更受临床医生的推崇^[31]^。但若病灶中实性变的比例超过25%，肺叶切除仍为首选^[30]^。而相比于手术，细针抽吸活检并不是GGO病变的首选，其原因主要有：①CT图像上的表现和病理学诊断之间的联系已被初步阐释，比如CT图像上的实变就与病理学上的微侵袭性肺腺癌表现出了一致性^[32, 33]^，并且侵袭性肺腺癌中的贴壁亚型（lepidic）肺腺癌也可在CT图像上表现为GGO^[34]^；②细针抽吸活检可能导致并发症^[35]^；③电视辅助胸腔镜手术（video-assisted thoracoscopic surgery, VATS）对患者造成的创伤很小^[4]^。在GGO结节的随访方面，Gould等于2013年在美国胸科医师协会第三版指南中指出，对于直径≤5 mm的纯GGO结节（不含实性成分），不建议进一步检查评估；而对于直径 > 5 mm的纯GGO结节（不含实性成分），建议每年一次胸部CT随访，至少随访3年。对于直径≤8 mm的部分实性GGO结节，建议在第3、12、24个月分别胸部CT随访一次，并于之后的1年-3年内每年随访一次；而对于直径 > 8 mm的部分实性GGO结节，建议每3个月随访一次胸部CT，并进行进一步的正电子发射计算机断层显像（position emission computed tomography, PET）检查、非手术性活检，以及对一直存在的结节行手术切除^[36]^。

## 6 总结

GGO以其独特的CT表现及其与肺部恶性病变的联系而迅速成为肿瘤科和胸外科领域的研究热点，在临床工作中，临床医生应当对CT上表现出GGO或GGO结节的病例进行长期随访，并且在必要的时候（如病灶 > 15 mm、病灶中实性变的成分 > 5 mm、病灶在随访过程中明显增大2 mm以上）对其进行手术干预，手术应当尽可能选择局限性手术，以更大程度地保留患者的肺功能，避免过度诊疗；对实性变占25%以上的CT上表现为GGO的病灶，应当考虑标准化肺叶切除术。同时，临床医生应当加强对GGO的认识，增强对GGO恶变的警惕性，防患于未然。笔者也认为，在诊治患者时要密切注意患者心理的变化，如果患者对GGO的表现过于紧张，则要抚平其紧张的情绪，必要时哪怕其未达到手术指征，但若其强烈要求，则也可对其进行手术，重视医学、心理、社会三者之间的联系，对不同的患者采取不同的对策。最后，笔者期待将来会有更多的临床工作者和科研工作者来研究这一课题，用更多的临床数据和实验结论来找到循证医学证据，使GGO的诊治更加规范化，并充分考虑不同患者的需求，针对其自身情况制订具体的诊疗方案，实现个体化医疗和精准医疗，为更多的肺癌患者和潜在的肺癌患者带来福音。
